# Supplementary material for: Restoring oak forests through direct seeding or planting: Protocol for a continental-scale experiment
Source: PLoS One. 2021 Nov 4;16(11):e0259552. doi: 10.1371/journal.pone.0259552 (PMC8568285; doi:10.1371/journal.pone.0259552)
Supplement: S1 Fig — (PDF) [file pone.0259552.s001.pdf]

Restoring vegetation through direct seeding or planting: A continental-scale experiment

The experiment

Collaborative field mini-experiment that explores the *seeding vs. planting* dilemma in ***Quercus* spp**. along environmental gradients through a network of participants, aiming to contribute to knowledge for improving forest and agroforestry ecosystem restoration.

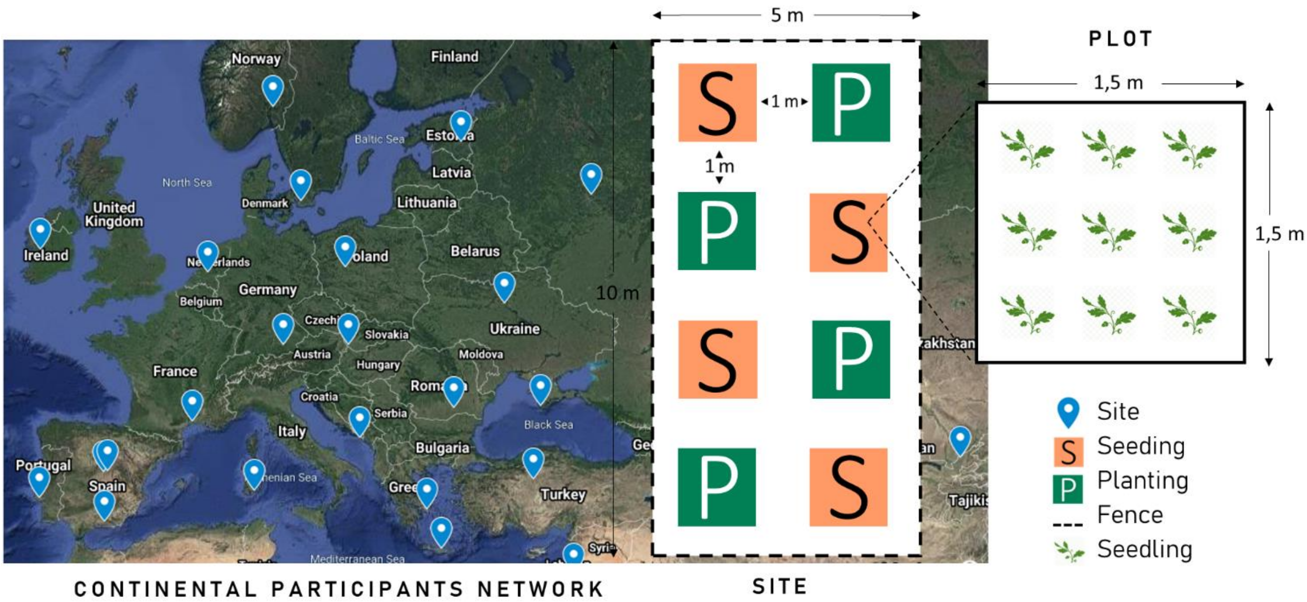

*site locations are hypothetical; for agreed participants see link above

How will we do it?

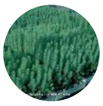

Participants will **establish a site** and select **local oak** species. One participant/ species allowed.

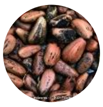

Participants will follow a standardised **protocol** for **seeding and planting**...

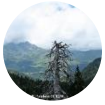

... and **measure** seedling performance.

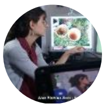

Through **meta-analysis**, we will **analyse the data** and publish the results collaboratively.

- **Autumn 2021 – 2024**
- Replicated across the **continent**
- **Open** to propose new sites
- **Low-cost** and easy-to-implement
- **Voluntary participation**
- **Self-financed**
- **More info** in June 2021

Join in and participate!

Register your interest here

Email: seedvsplant@gmail.com

UNIVERSIDAD DE GRANADA · Facultad de Ciencias · IISTA · PEN-CAFoRR · COST EUROPEAN COOPERATION IN SCIENCE & TECHNOLOGY · MINISTERIO DE CIENCIA E INNOVACIÓN · UNIÓN EUROPEA FONDO EUROPEO DE DESARROLLO REGIONAL · AGENCIA ESTATAL DE INVESTIGACIÓN
