# Supplementary material for: Restoring oak forests through direct seeding or planting: Protocol for a continental-scale experiment
Source: PLoS One. 2021 Nov 4;16(11):e0259552. doi: 10.1371/journal.pone.0259552 (PMC8568285; doi:10.1371/journal.pone.0259552)
Supplement: S2 Fig — (PDF) [file pone.0259552.s002.pdf]

Restoring vegetation through direct seeding or planting:

A continental-scale experiment

What does a site look like?

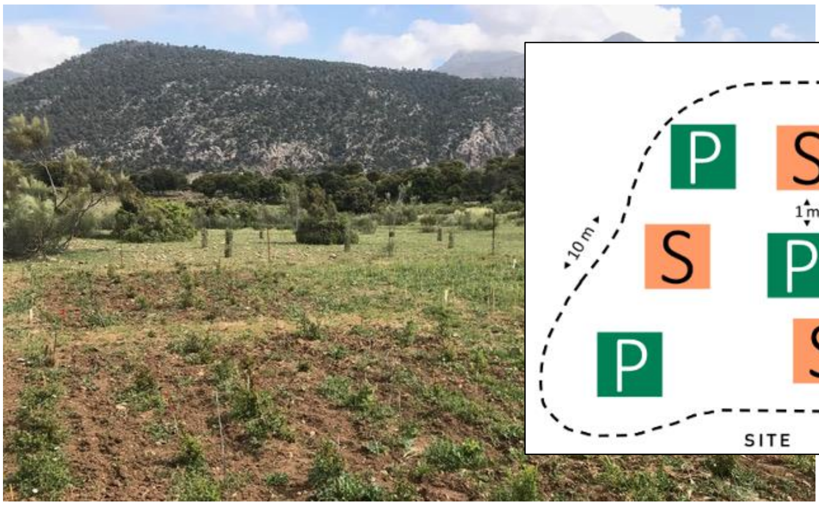

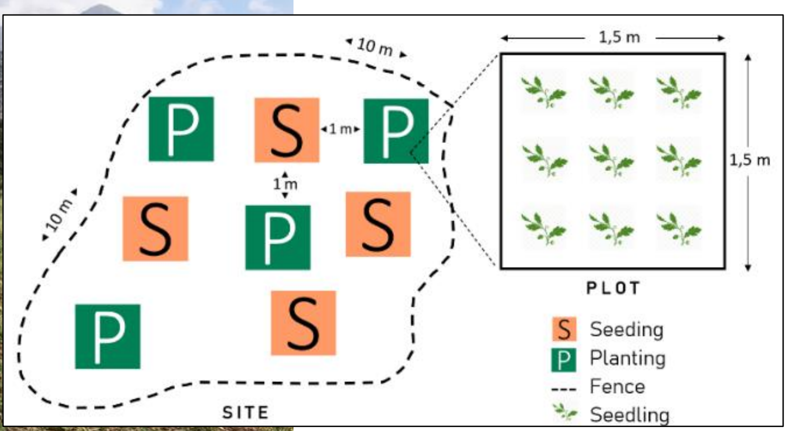

- Each **site** consists of 8 plots of 1,5 x 1,5m per species of *Quercus*.
- Each **plot** contains 9 plant-points with either **sown or planted seedling**.
- Target number of plants: 8 plots x 9 plants = **72 plants per species** (will be grown in excess to account for emergence and mortality).
- These 8 plots will comprise **4 replicates** of each of the 2 revegetation methods.
- # of species: **1 or more species /site**.
- Total # of plots = 8 plots x # of species.
- **1 participant allowed per site and species**.

Next steps

Have a look at the map!

Think about your local oak species and site location.

Any questions, please contact
seedvsplant@gmail.com

Complete protocol will be ready in autumn!

To take into account

- The **plots** must be max ~100 m from each other, have similar conditions and be at least 1 m apart.
- For a regular **site with one species**, the minimum size would thus be 5 x 10 m.
- The allocation of **revegetation method and species** to each plot will be made **randomly**.
- Plants must be **protected from herbivores**, through fences. We may send seed protectors vs. rodents.
- **Sites must be located on an open area that is otherwise suitable for the selected species**. Abandoned fields, clearcuts, burnt areas, cleared windthrows, and other open areas. However, highly degraded areas or sites with heavily altered soil, such as roadsides, mines, and polluted sites, will not be considered.
- The **conditions of the site** (soil type, bedrock, elevation, etc.) should be representative of the area.
- The site should be **as flat as possible**, max. 10% slope. Flat terraces on an otherwise steeper mountain side are ok.
- There should be **no trees or shrubs** inside the plots.
- Each plot must be clearly **identified and marked**.

UNIVERSIDAD DE GRANADA
Facultad de Ciencias
IISTA
PEN-CAFoRR
cost EUROPEAN COOPERATION IN SCIENCE & TECHNOLOGY
MINISTERIO DE CIENCIA E INNOVACIÓN
UNIÓN EUROPEA FONDO EUROPEO DE DESARROLLO REGIONAL "Una manera de hacer Europa"
AGENCIA ESTATAL DE INVESTIGACIÓN
