# Supplementary material for: Restoring oak forests through direct seeding or planting: Protocol for a continental-scale experiment
Source: PLoS One. 2021 Nov 4;16(11):e0259552. doi: 10.1371/journal.pone.0259552 (PMC8568285; doi:10.1371/journal.pone.0259552)
Supplement: S3 Fig — (PDF) [file pone.0259552.s003.pdf]

Restoring vegetation through direct seeding or planting:

A continental-scale experiment

SEEDING VS PLANTING
A collaborative continental experiment

Timing of the experiment

	ACTION	2021			2022			2023					2024		
		☀	🍂	❄	❀	☀	🍂	❄	❀	☀	🍂	❄	❀	☀	🍂
PARTICIPANTS	Site preparation		Soil preparation. Marking plots and plant-points	Wee-ding*	Weeding*	Weeding*		Soil preparation. Weeding*	Wee-ding*	Weeding*		Wee-ding*	Wee-ding*	Weeding*	
PARTICIPANTS	Seed preparation		Seed collection, selection, storage												
PARTICIPANTS	Seeding		Seeding in the field		Measure emer-gence			Initial seedling measurements		Seedling measurements after growing season		Assess survival after winter		Seedling measurements after growing season	
PARTICIPANTS	Planting		Start nursery cultivation					Outplanting nursery seedlings & Initial seedling measurements. Dead-seedling replacement & measurement		Seedling measurements after growing season		Assess survival after winter		Seedling measurements after growing season	
PARTICIPANTS	Deliverables		Send site and species info. Post seeds and soil		Data #1			Data #2		Data #3		Data #4		Data #5	Post dry plants (to be decided)
COORDINATORS	Germination test and soil analysis		Start of germination test	Soil analysis	End of germina-tion test										
COORDINATORS	Data management	Attract and inform partici-pants	Disseminate full study protocol			Prepare emergence, soil and germination data			Prepare data		Prepare data			Prepare and analyse data	
COORDINATORS	Write-up			Write up introduc-tion & methods				Update introduction & methods						Prepare results & discussion	

*when ~80% of the plots is covered by herbs

Material requirements

The experiment is non-funded.
Participants will need:

- Local seeds
- Site for planting
- Greenhouse or a nursery facility for plant cultivation
- Transportation of containerized seedlings
- Materials for digging, marking plots and tagging plants
- Oven, lab & field scales
- Ruler & caliper

Deliverables

Beginning
* Seeds for germination test
* Composite soil sample

End
* Oven-dried plants

Along the experiment
Data and information
- Species selection
- Site selection
- Data

Next steps

Register if you haven't done so

Have a look at the map!

Complete protocol will be ready in autumn.

Precise site locations and species to be requested soon

Any questions, please contact
seedvsplant@gmail.com

UNIVERSIDAD DE GRANADA · Facultad de Ciencias · IISTA · PEN-CAFoRR · cost EUROPEAN COOPERATION IN SCIENCE & TECHNOLOGY · MINISTERIO DE CIENCIA E INNOVACIÓN · UNIÓN EUROPEA FONDO EUROPEO DE DESARROLLO REGIONAL "Una manera de hacer Europa" · AGENCIA ESTATAL DE INVESTIGACIÓN
