# Supplementary material for: Restoring oak forests through direct seeding or planting: Protocol for a continental-scale experiment
Source: PLoS One. 2021 Nov 4;16(11):e0259552. doi: 10.1371/journal.pone.0259552 (PMC8568285; doi:10.1371/journal.pone.0259552)
Supplement: S4 Fig — (PDF) [file pone.0259552.s004.pdf]

Restoring vegetation through direct seeding or planting:

A continental-scale experiment

Acorn collection

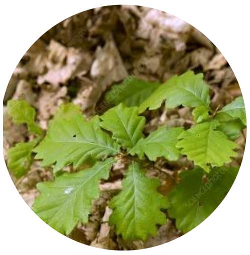

Target species

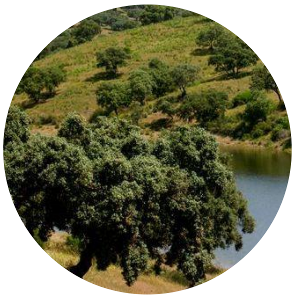

Identification of a source population

- Minimum 10 parent trees
- Local populations growing at similar elevation, aspect and substrate and near the experimental site.
- Collect ripe acorns and from the tree if possible

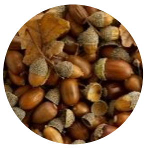

Seed collection

- Similar # of seeds / parent tree
- Needed: 72 seeded + 72 nursery-grown + 150 germination test = 294 <u>sound</u> acorns. Recommended: **minimum 500 acorns**

To take into account

- If necessary, local seeds may **exceptionally be bought**.
- Acorn **selection:** flotation method.
- Acorn **storage:** in zip polyethylene plastic bags 50 µm thick (which we will send you) → refrigerator 1 –4 ºC. Avoid stacking bags!
- **Subsequent use** of the seeds should occur as soon as conditions allow it (within weeks).
- Soon after collection, a **parcel** should be posted to Granada containing 150 seeds and a composite soil sample from the experimental site (more info soon!).

Next steps

Have a look at the map!

Any questions, please contact seedvsplant@gmail.com

Fill in the attached site form.

Full protocol available by next week.
