# Supplementary material for: Restoring oak forests through direct seeding or planting: Protocol for a continental-scale experiment
Source: PLoS One. 2021 Nov 4;16(11):e0259552. doi: 10.1371/journal.pone.0259552 (PMC8568285; doi:10.1371/journal.pone.0259552)
Supplement: Attachment — Submitted filename: Review-PO.docx [file pone.0259552.s005.docx]

Dear Teresa Morán-López

The manuscript "Restoring vegetation through direct seeding or planting: Protocol for a continental-scale experiment" provides a clear and detailed methodology to test how seeds and seedlings interact with a range of environmental conditions across Eurasia. This information is important because it can guide future restoration efforts and make them more successful and cost-efficient.

The protocol is clear and well-written. The authors provide all the information needed to replicate the experiment across multiple sites and provide an overview of the data analysis that will be used and how the data will be make available.

I find the protocol very useful and I am looking forward to see their results to understand better how trees species and environmental parameters interact.

I only have two comments regarding the protocol and I think if the authors specify these things further, the experiment and data will be more robust. Please see comments below:

1. I think it would be better if the authors select a couple of insecticides that will be allowed to be used for the experiment. There are lots of products out there and if each participant chooses what they think is best, they might be adding another source of variation. I would suggest the authors choose one or two insecticides that could be applied if needed.

2. Thinking about reducing variation, I would also suggest to choose one or two species of Quercus to be used across the study sites. We know we can find different physiological responses from different individuals of the same species, so opening the variation to a whole genus could make the drawing of conclusions harder. What about using *Quercus robur* and/or *Q. petraea*? If the authors choose the same species, they could also collect very interesting data about the different responses of the same species to the range of environmental conditions present across their study sites.

Overall I enjoyed reading the protocol and I think this experiment is a great idea to test on a continental scale.
