# Supplementary material for: Restoring oak forests through direct seeding or planting: Protocol for a continental-scale experiment
Source: PLoS One. 2021 Nov 4;16(11):e0259552. doi: 10.1371/journal.pone.0259552 (PMC8568285; doi:10.1371/journal.pone.0259552)
Supplement: Attachment — Submitted filename: Response to Reviewers.docx [file pone.0259552.s007.docx]

Dear Dr. Morán,

Thank you very much for your time, the positive feedback, and the opportunity to improve our study protocol with your and the reviewers’ suggestions. I hereby submit an improved version of our protocol, in which we have followed most of the advice provided, as well as a letter with a detailed point-to-point response to each comment.

We highlight that the suggestion made by Reviewer 2 on addressing bias has occupied many of our thoughts and discussions in the last months. In fact, some of us recently published an opinion article ([doi:10.1111/rec.13372](https://doi.org/10.1111/rec.13372)) on the difficulty in establishing *ceteris paribus* conditions to compare seeding and planting as regeneration methods and we are addressing the trade-offs among multiple possibilities in an ongoing field experiment in Granada (see Table 1 in the above-cited paper). For our proposed continental-scale experiment, we consider it essential that the seeded and planted individuals be of the same age and origin, as otherwise we would risk concluding e.g. that older seedlings are taller than younger ones. This has the additional advantages of being able to use the same seed lot for both methods and avoiding the risk of viability loss of acorn batches stored for long time. Although this comes at the cost of seeding one year prior to planting –and therefore conditions may vary among years–, we expect this to produce only random heterogeneity in our dataset because we will be using many different sites (and this heterogeneity will be modelled in our analyses with site-specific random effects); the study could indeed face the risk of systematic bias, as suggested by the reviewer, in case it was restricted to one site. Among the multiple possible ways of conducting our experiment, we therefore consider that the way in which plan to conduct it is the most robust way possible.

Please find detailed replies to all comments below. Thank you again for your consideration and particularly for your prompt handling of our manuscript, which has already helped us to communicate the research design with the participants.

Sincerely,

Alexandro Leverkus (on behalf of all authors)

P.S. As requested in your decision email, we wish to add the following to our financial disclosure statement: “*The funders had no role in study design, data collection and analysis, decision to publish, or preparation of the manuscript”*

**Editor comments**

*Dear Dr. Leverkus,*

*I have now received the comments from both reviewers. All of us are very excited about this large-scale experiment that aims to compare the effectiveness of seed sowing vs planting seedlings in revegetation plans. Both reviewers agree that the study is timely and the protocol is well-described. Also they provide some interesting advices that you may want to incorporate.*

*Reviewer 1 provides some advices about the use of insecticides and limiting the experiment to a lower number of species. I guess, you have decided to not constrain the number of species in order to take into account the natural variabililty found in Eurasia. This would  allow to provide general guidelines. However, in the analyses section it could be highlighted that the meta-analyses will take into account species-specific effects (probably as a random factor). Now it is stated along other potential covariates in L446.*

🡪Indeed, the plan is to produce relevant outcomes in terms of species and spatial distribution. Allowing any native oak species is also inclusive for participants from most parts of the continent.

Thank you for the suggestion. We indicate in the Statistical analyses section that we aim to model the effect of species, of species’ traits, and of the phylogenetic relatedness among species.

*Reviewer 2 is concerned about sowing seeds and planting seedlings in different years. I understand your reasoning (this way one-year-old seedlings are grown in the same environmental conditions), but also that this could make comparisons difficult if environmental conditions strongly vary between years. Could you provide a little bit more details about this issue?*

🡪Comparing seeding and planting involves choosing among multiple possible sources of heterogeneity or bias. We have chosen to conduct the treatments in different years because, although weather differences can affect outcomes locally, such effects are unlikely to result in systematic bias in our continental-scale dataset, and they can be modelled as part of the random cross-site heterogeneity. The advantages of our approach are that we will homogenize seedling age and seed origin, and we avoid the need for additional infrastructure –and associated risks– such as refrigerators at ~50 sites. Please find a more detailed response below.

*Finally, since your current deadline is October i suggest to move it some weeks (if possible) so that the paper is published before. I will be willing to accept it as soon as possible.*

🡪We are open to accepting new participants as long as they are able to implement the experiment in time, which is greatly dependent on the phenology of trees at each location. We now indicate this in the revised version of the protocol.

We are grateful for your efforts in promptly handling our manuscript.

**Reviewers' comments:**

***Reviewer #1:*** *Dear Alexandro,*

*Your manuscript "Restoring vegetation through direct seeding or planting: Protocol for a continental-scale experiment" provides a clear and detailed methodology to test how seeds and seedlings interact with a range of environmental conditions across Eurasia.*

*The protocol is clear and well-written. You and your co-authors provide all the information needed to replicate the experiment across multiple sites and provide an overview of the data analysis that will be used.
I find the protocol very useful and I am looking forward to see your results to understand better how trees species and environmental parameters interact.*

🡪We very much appreciate your time and feedback. Please find responses below.

*I only have two comments regarding the protocol and I think if you specify these things further, your experiment and data will be more robust. Please see comments below:*

*1. I think it would be better if you* ***select a couple of insecticides*** *that will be allowed to be used for the experiment. There are lots of products out there and if each participant chooses what they think is best, you might be adding another source of variation. I would suggest you and your co-authors choose one or two insecticides that could be applied if needed.*

🡪Thank you for the suggestion, which we have discussed. But given the large variety of potential pests, available insecticides, and regulations across sites, we have decided not to indicate particular products at this stage. We have, however, indicated that the suitability of applying a particular product must be agreed on with the organizers, which should give room to investigate if the given product could produce unwanted side effects and propose alternatives.

*2. Thinking about reducing variation, I would also suggest you* ***choose one or two species of Quercus*** *to be used across the study sites. We know we can find different physiological responses from different individuals of the same species, so opening the variation to a whole genus could make the drawing of conclusions harder. What about using Quercus robur and/or Q. petraea? If you choose the same species, you could also collect very interesting data about the different responses of the same species to the range of environmental conditions present across your study sites.*

🡪Thank you for the suggestion. We have gone through multiple versions of the protocol, including proposing different combinations of species, and opted for narrowing it down to oaks because of a) their wide distribution, b) their wide use for forestation, and c) oaks being at the core of the seeding vs. planting dilemma due to their large-sized seeds. Restricting the experiment to some species, as suggested, could potentially help gain more in-depth knowledge about those species, but it would unfortunately reduce the scope of our study to particular species and to their distribution areas, as well as reduce the amount of data because it would exclude many current participants.

We do however consider that the point raised is of great relevance, and we aim to address it by a) seeking to ideally have at least 3 sites for each species, for which participants are asked to provide a list of potential “plan B” species in case one of their “plan A” species is not used anywhere else; and b) modelling the effect of species in statistical analyses.

*Overall I enjoyed reading your protocol and I think is a great idea to test on a continental scale.*

🡪Thank you very much for your insights!

***Reviewer #2****: PLEASE SEE ATTACHED DOCUMENT FOR SPECIFIC COMMENTS*

🡪Thank you very much for the comments; we have followed the suggestions in the improved version.

*The paper is well written and clear, and the topic is very interesting. I’m sure that the outputs of this multi experiment will contribute effectively to optimize the results of forest planting. Congratulations to the authors.*

🡪We very much appreciate your time, positive feedback, and suggestions for improvement. Please find detailed replies below.

*But I must focus the attention on the question of the bias control. Authors propose that sowing must be conducted one year before than planting while seedlings of the same seedlot grow in the nursey. This avoid seeds coming from different genetics. However, this mean that sowing and planting will be conducted in different years. Authors argue that this schedule provides comparison performance of the same age seedlings. However, planting and sowing at different years can lead to huge inter-annual differences in environmental conditions. If the study aims at linking environmental conditions of the planting site with size effect of planting versus direct seeding, then comparing performance in two different years is not very appropriate. In many environments, inter-annual differences can be larger than inter-sites one.*

🡪We appreciate the comment, as it pinpoints a major aspect on how to properly design this kind of experiment. In fact, it has been our frequent topic of discussion in the last years, which led to the recent publication of an opinion article on how bias can be handled when comparing seeding vs planting (Leverkus et al. 2021 Restor. Ecol. doi:10.1111/rec.13372. There seems to be no perfect way of doing it, as any option involves trade-offs among multiple possible sources of bias. We have opted for planting one year after seeding for the following reasons:

1. It avoids genetic differences between the two methods because the same seed batch is used for both methods.

2. It removes bias from having planted vs. seeded individuals of different ages. In fact, this makes statistical analyses much more straight-forward –for instance, comparing height growth of newly germinated seedlings with that of 1-yr old planted seedlings would be quite challenging. In our experiment, seeded and planted individuals will be of the same age and the effect of revegetation method on their performance can thus be assessed independently of age differences.

3. Due to the multi-site nature of our experiment, weather differences between years are not expected to introduce systematic bias in our dataset. This means that, whereas weather differences can indeed affect the outcomes of the experiment at particular sites, the direction and magnitude of this effect can reasonably be expected to vary across sites, as they will be scattered across the continent. Such variability can be properly handled by our proposed statistical models, where there is a random component related to each site that considers heterogeneity in treatment effect sizes. In the case of a single-site experiment we do see the problem of potential bias, as the conditions of each year can produce a confounding effect with that of treatment.

*Besides, the mentioned “siblings” are quite different in terms of root architecture, nutrients, SLA, dry mass, carbohydrate reserves and so on.*

🡪 Yes indeed. These are the differences that are produced by the two revegetation methods, and they are the drivers of the effects that we expect to measure. This is the reason why we plan to measure the seedlings of both treatments at the time of outplanting, so we have initial values that help set the baseline and that can potentially be used as covariates to better understand how seedlings respond to revegetation method.

Following this comment, we have added to the protocol that some of the seedlings that will not be used in the experiment –i.e., those discarded during seedling selection as described in the protocol– will be used to measure initial biomass.

*Those differences plus environmental ones from one year to another could be higher than argued differences of age. Optimal solution is using the same seed lot but storing the acorns for one year to sow them in the second year along with planting. See for instance: Oliet, J.A.; Vázquez de Castro, A.; Puértolas, J. 2015 Establishing Quercus ilex under Mediterranean dry conditions: sowing recalcitrant acorns versus planting seedlings at different depths and tube shelter light transmissions New Forests 46: 869-883*

🡪 We acknowledge that this can be a good option in some cases –we are, in fact, testing this and other alternatives in a field experiment in Granada. The problem that we see in applying it to the proposed continental-scale experiment is that a) the storage of seeds would require additional infrastructure at all ~50 sites, b) seeds may lose viability during 1-yr storage if not properly stored, thereby producing systematic bias because it would reduce the performance of the seeding treatment across all sites, and c) the magnitude of the loss of seed viability would likely differ across sites, thereby introducing heterogeneity in this bias. The way in which we propose the experiment avoids these problems and allows to conduct one single germination test that will be applicable to both revegetation treatments.

Despite this, we see the value in the alternative approach proposed and are planning to propose a voluntary extension of the experiment with this additional, third treatment.

🡪Thank you again for your time and thoughtful insights!
